# Rootstock Affects the Fruit Quality of ‘Early Bigi’ Sweet Cherries

**DOI:** 10.3390/foods10102317

**Published:** 2021-09-29

**Authors:** Valter Martins, Vânia Silva, Sandra Pereira, Sílvia Afonso, Ivo Oliveira, Marlene Santos, Carlos Ribeiro, Alice Vilela, Eunice Bacelar, Ana Paula Silva, Berta Gonçalves

**Affiliations:** 1 Centre for the Research and Technology of Agro-Environmental and Biological Sciences (CITAB), INOV4AGRO, University of Trás-os-Montes e Alto Douro, UTAD, Quinta de Prados, 5000-801 Vila Real, Portugal; valterm@utad.pt (V.M.); vaniasilva@utad.pt (V.S.); safonso@utad.pt (S.A.); ivo.vaz.oliveira@utad.pt (I.O.); mpsantos@utad.pt (M.S.); areale@utad.pt (E.B.); asilva@utad.pt (A.P.S.); bertag@utad.pt (B.G.); 2 Department of Agronomy, University of Trás-os-Montes e Alto Douro, UTAD, Quinta de Prados, 5000-801 Vila Real, Portugal; cribeiro@utad.pt; 3 Chemistry Centre (CQ-VR), University of Trás-os-Montes e Alto Douro, UTAD, Quinta de Prados, 5000-801 Vila Real, Portugal; avimoura@utad.pt

**Keywords:** cherry quality, rootstocks, sensory profile, scion–rootstock combination, *Prunus avium* L.

## Abstract

The cherry rootstock influences the performance of the scion cultivar. It has an effect on cherry fruit quality, tree growth, yield and yield efficiency and floral and foliar nutrition. In this work, the influence of Saint Lucie 64 and Maxma 60 rootstocks on the fruit quality traits of cv. Early Bigi was evaluated. For this, several parameters, namely fruit weight (FW) and size (FS), soluble solids content (SSC), pH, titratable acidity (TA), flesh firmness (FF), epidermis rupture force (ERF), color and sensory profile, were assessed. Results showed that the fruits from trees on Saint Lucie 64 presented higher FF and ERF values and, consequently, better texture. On the other hand, fruits from trees on Maxma 60 showed sweeter cherries (higher SSC). Moreover, these trees presented the darkest cherries (lower values of L*, a*, b*, C* and *hue*°) and the highest SSC. Therefore, although the trees on Saint Lucie 64 produced firmer cherries, it was those with the Maxma 60 rootstock that produced sweeter and darker fruits. In conclusion, both scion–rootstock combinations proved to be good options for the region of Resende.

## 1. Introduction

The sweet cherry tree (*Prunus avium* L.) belongs to the Rosaceae family and is a crop of great economic importance [1]. Sweet cherry is a fruit with exceptional organoleptic and nutritional qualities and recognized health benefits, associated with its health-promoting compounds [2,3,4]. Indeed, cherries are rich in C, A, E and K vitamins and phenolic compounds [5] such as flavonoids, flavan-3-ols and flavonols and non-flavonoid compounds such as hydroxycinnamic and hydroxybenzoic acids that exhibit high antioxidant activity [6,7,8,9]. Polyphenol intake is associated with decreased risk of cardiovascular disease, arthritis, and neurodegenerative disorders [10] and prevents oxidative stress-induced disorders such as intestinal inflammation disorders and neuronal cell death [11], as well as cancer risk [12]. Beyond that, sweet cherry consumption has an anti-inflammatory [13,14], antioxidant and antitumor activity [9,15] helping to lower blood pressure, control body weight and diabetes and prevent Alzheimer’s disease [16].

The worldwide growing interest in this culture and for quality cherries has led to significant improvements in their production and marketing over time [17]. There are several rootstocks for cherries worldwide from different breeding programs. These have focused mainly on improving certain characteristics, such as yield, nutritional content, flowering and ripening time [1], taste, fruit size, firmness and color, precocity and resistance to fruit cracking and diseases [18], which led to the appearance of a large number of sweet cherry rootstocks [1].

For solving the limiting factors of production and conditioning of market demands on productivity, a short juvenility period and high fruit quality, the rootstock selection has gained great importance [19]. Thus, choosing the most appropriate sweet cherry rootstock proved to be essential, the priority being given to their compatibility with the majority of cultivars in use as well as the adaptability to different agro-ecological conditions [20]. An ideal rootstock should provide a better anchorage and induce good tree survival, high annual yields and acceptable fruit color and size [21,22]. Rootstocks should also restrict the scion vigor in order to enable the tree to adapt to adverse soil conditions (pH, drought, texture, drainage) as well as to increase tolerance to biotic (nematodes, insects, diseases) and abiotic (e.g., water deficit, salinity, low temperatures) stress factors [23].

The rootstock selection at the time of installation of a new orchard is dependent on several factors, namely the soil structure, texture and fertility, and the desired training system [18]. In modern orchards, vigor-reducing rootstocks have gained an advantage over the vigorous [24,25].

Although the fruit quality depends essentially on the scion genotype, it can also be influenced by the rootstock [26,27,28] and also by edaphoclimatic conditions. Thus, the assessment of rootstock influence on productivity [29] and on fruit quality [30] is of extreme importance. There are several studies that reported the influence of the scion × rootstock combination on cherry fruit quality [31,32,33,34,35]. Kader [36] defined fresh fruit quality factors, hygiene, appearance (size, weight, shape), texture (firmness, hardness/softness), flavor (sweetness, sourness) and nutritional factors. However, quality criteria vary from consumer to consumer, and consumer preferences dictate the choice of fruits [37].

Several studies reported that rootstock affects the tree vigor [38], vegetative growth, and yield efficiency of grafted cultivar [35,39,40]; productivity index [41]; fruit quality parameters such as size [42], firmness [43], soluble solids content, acidity, color and taste [42,44]; precocity; and resistance to fruit cracking and diseases [45]. In fact, with the right combination of scion × rootstock, it is possible to obtain fruits with higher levels of firmness, weight, sugars, vitamins and phenolic compounds that boost the fruit antioxidant activity [46]. 

However, the scion–rootstock combination is not always compatible, and cases of genetic incompatibility with the scion graft may occur [18,47]. Feucht and Treutter [48] reported that the incompatibility of grafted fruit plants is a phenomenon of premature senescence caused by physiological and biochemical processes. Some incompatibilities have been reported by several authors; for example, Usenik and Štampar [49] found that cv. ‘Lapins’ showed low compatibility with F 12/1, Gisela 5 and Weiroot 158 rootstocks, which resulted in a pronounced accumulation of polyphenols (*p*-coumaric acid) above the graft union as a stress response to grafting. Later, in 2006, the same was found for apricot cultivars grafted on heterospecific rootstocks [50]. Mng’omba et al. [51] also reported graft incompatibility of loquat; they observed the formation of calluses above the union resulting from the production of *p*-coumaric acids and anthocyanins.

As it was reported before, there are different rootstocks that can induce differences in plant growth and yield. Cherry rootstocks exert a remarkable influence on the behavior of the grafted variety, since the vegetative development, harvest date, yield, fruit quality and tree mineral nutrition are determined by rootstocks [52]. Thus, within the scope of our work, it is important to know the rootstocks (Maxma 60 and Santa Lucia) as well as the cultivar under study (cv. Early Bigi). The Maxma series results from the crossing of *Prunus avium* and *Prunus mahaleb* and combines good productivity with good soil adaptation. Maxma 60 rootstock usually presents an erect growth, high vigor, semidwarfing behavior and high compatibility with various cultivars. It also shows resistance to *Phytophthora cambivora* and *P. megasperma* infections, great adaptability to several types of soils and less favorable climates, early production and high resistance to oxygen restriction in compact soils but low drought resistance [53]. On the other hand, Saint Lucie 64 clone (SL 64), also called *Prunus mahaleb*, a traditional rootstock, is compatible with a large number of varieties and presents reduced vigor, faster installation and production and a good adaptation to different types of soil [54]. However, due to its lightly branched deep roots, it requires good drainage, since it is sensitive to root asphyxiation caused by compact soils. It is also a rootstock with high drought resistance, tolerant to frequent ferric chlorosis of calcareous soils of the Mediterranean region [54]. According to Jimenez et al. [55], trees grafted on SL 64 rootstock have an earlier and higher productivity than trees grafted on Maxma 60, while trees grafted on Maxma 60 present fruits with higher soluble solids content and sugars. 

Early Bigi cultivar was selected in France, presenting early flowering and maturation and thick and rounded fruits with dark red skin and good pulp with medium firmness but little crack resistance [56,57].

Knowing that the choice of the correct scion–rootstock combination for each growing region is crucial to improve the productivity and quality of sweet cherries, the objective of this study was to evaluate the influence of two different rootstocks, ‘Sant Lucie’ (SL 64) and ‘Maxma 60′, on the quality parameters (sweet taste, color, acidity, firmness) and sensorial perception of fruits of sweet cherry cultivar ‘Early Bigi’ and to find correlations between all the evaluated parameters. Thus, several parameters, namely fruit weight (FW), fruit size (FS), soluble solids content (SSC), pH, titratable acidity (TA), flesh firmness (FF), epidermis rupture force (ERF) and color, were quantified and a sensory profile was obtained as a result of a sensory proof. This study is of extreme importance since there is a great lack of knowledge about the adaptation of the many scion–rootstock combinations available to the Portuguese edaphoclimatic conditions.

## 2. Materials and Methods

### 2.1. Experimental Design and Sweet Cherry Raw Material

The trial was carried out in an orchard located in Alufinha, S. João de Fontoura, municipality of Resende (Viseu district), north of Portugal (latitude 41°12′ N, longitude 7°93′ W, altitude 149 m). Ten 10-year-old sweet cherry trees from cv. Early Bigi grafted on both studied rootstocks (SL 64 and Maxma 60) were selected. Trees were spaced 3.0 m between rows and 2.5 m from each other in the row. Fruits from trees of SL 64 rootstock were hand-harvested on 2 May 2019, and fruits from trees of Maxma 60 rootstock were harvested on 10 May 2019. All trees were drip-irrigated and fertilized according to the owner’s guidelines. Afterward, two sublots of cherries without defects were prepared, one for the sensory analysis and the other for the biometric assays.

Weather conditions, namely precipitation (mm) and mean temperature (°C), in the year 2019, were recorded by a meteorological station placed in São João de Fontoura, near the experimental site. Records showed that 2019 was an extremely hot and dry year. The mean temperature ranged from 7.8 °C in January to 22.3 °C in July. Precipitation values were relatively low throughout the year, with a peak of precipitation being recorded in the last 3 months of the year, reaching its maximum in November with 616.4 mm.

### 2.2. Sensory Profile

Fruits from the first sublot were used to evaluate the sensory profile of sweet cherries. For this, 15 cherry attributes (appearance, epidermis softness, color intensity, color uniformity, peduncle color, odor intensity, sweet taste, acidic taste, bitter taste, astringency, strange taste, cherry flavor, strange flavor, firmness and succulence) adapted from Chauvin et al. [58] were evaluated by 12 participants aged between 35 and 50 years old. The sessions were kept at room temperature in a sensory laboratory with individual booths for each panelist [59]. Attribute intensities were scored on a five-point scale, ranging from 1 (lowest intensity) to 5 (highest intensity) [60,61]. Two sweet cherry fruits of each rootstock were presented to each taster. Samples were randomly presented to the panelists, coded with a three-digit code number. Samples were placed on a white pyrex plate and at room temperature for 2 h before each session to obtain a temperature of about 18 °C. Panel participants were required to clean their palates with a sip of water before each sample. Participants were nonsmokers and avoided use perfume and drinking or consuming food that could affect their performance 1 h before tasting [62].

### 2.3. Fruit Weight (FW) and Size (FS)

The weight and size of 30 fruits of the second sublot were determined. Fruit weight (g) was determined using an electronic balance (EW2200-2NM, Kern, Balingen, Germany), and fruit size (mm) (height, and smaller and larger diameter) was measured using a digital caliper (Mitutoyo, Hampshire, UK).

### 2.4. Fruit Color

Fruit color was measured on two opposite sides of the same 30 fruits used to determine weight and size, with a chromameter (CR-300 Minolta, Tokyo, Japan) expressing the color as L*, a* and b* values. Cherry color was reported as chroma (C*) according to the formula C* = (a*2 + b*2)^1/2^ [63,64]. The L* coordinate indicates brightness varying from 0 (completely opaque or black) to 100 (completely transparent or white). A positive value of a* indicates redness (−a* is green), and a positive value of b* indicates yellowness (−b* is blue) in the hue circle [65,66]. The *hue* angle was calculated using the formula *hue*⁰ = arctg (b*/a*) and expresses the color nuance [66], and values are defined as follows: red-purple, 0°; yellow, 90°; bluish-green, 180°; and blue, 270° [67]. Results were presented as the average of 60 measurements (2 per fruit) with indicated standard deviation (SD).

### 2.5. Epidermis Rupture Force (ERF) and Flesh Firmness (FF)

Subsequently, ERF (N) and FF (N.mm^−1^) were determined, in the same 30 fruits, using a TA.XTplus texture analyzer (Stable Micro Systems, Godalming, UK) employing a 50 N load cell and a cylindrical probe with a diameter of 2.0 mm. The maximum force compressing 5 mm was measured at a speed of 1 mm·s^−1^ [62]. 

### 2.6. Total Soluble Solids, Titratable Acidity, Maturity Index, and pH 

The same 30 fruits from previous measurements were then divided into three groups of 10 fruits each. The juice of these fruits was extracted with an electrical extractor (ZN350C70, Tefal Elea, Zhejiang, China) for 1 min. The soluble solids content (SSC, in °Brix) was determined using a digital refractometer (PR-101, Atago, Tokyo, Japan). The juice pH was assessed with a pH meter (Jenway 3310). The titratable acidity (TA, g malic acid 100 g^−1^ of fresh weight) was determined by diluting 10 mL of juice with 10 mL of distilled water and titrating with 0.1 mol L^−1^ sodium hydroxide (NaOH) to pH 8.2 using an automatic titrator (Schott Easy Titroline). The maturity index (MI) was expressed as the ratio of SSC and TA and expressed as the mean of three repetitions with indicated standard deviation (SD) [62].

### 2.7. Statistical Analysis

Statistical analysis was performed using Software SPSS V.25 (SPSS-IBM, Corp., Armonk, New York, NY, USA). Statistical differences were evaluated by one-way analysis of variance (ANOVA) followed by Tukey’s *post hoc* multiple range test (*p* < 0.05).

Principal component analysis (PCA) was also performed in the same software. PCA is mostly used as a tool in exploratory data analysis and for making predictive models. PCA was performed by eigenvalue decomposition of the data correlation (Corr-PCA) matrix after normalizing the data matrix for each attribute.

Additionally, a discriminant analysis (DCA) was also performed using SPSS software to understand in which variables the effect of the rootstock was more pronounced.

## 3. Results and Discussion

Several studies have demonstrated that rootstock influences the performance of the grafted sweet cherry cultivar. There have been numerous reports of a relationship between cherry rootstocks and water relations, leaf gas exchange, mineral uptake, plant size, blossoming, fruit bud survival, fruit quality and yield efficiency [43,68].

### 3.1. Sensory Profile

**Figure 1:**
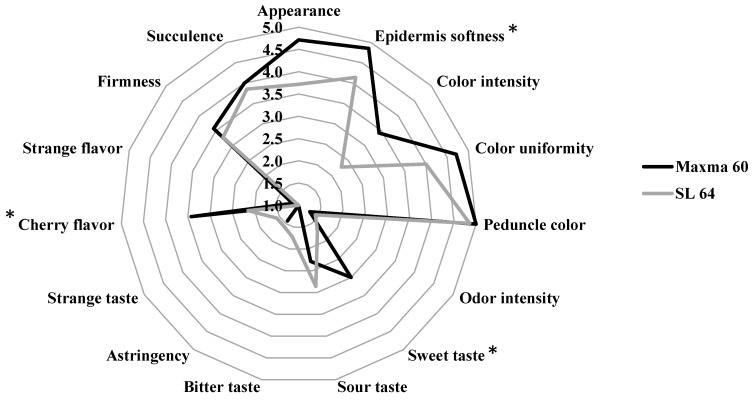
Sensory profile of sweet cherry cv. Early Bigi grafted on SL 64 and Maxma 60 rootstocks. * indicates statistically significant differences (*p* < 0.05) between rootstocks for the respective attribute, according to Duncan’s test.

The sensory profile of sweet cherry cv. Early Bigi grafted on SL 64 and Maxma 60 rootstocks is presented in Figure 1. In the sensory evaluation, significant differences were observed in only 3 of the 15 attributes evaluated: epidermis softness, sweet taste and cherry flavor. According to the panel of tasters, Maxma 60 rootstock presented softer epidermis, sweeter taste and flavorful cherries. 

These results were corroborated by the other evaluated parameters, namely maturity, chromatic and texture parameters. Indeed, the higher MI and the lower values of the chromatic parameters observed in fruits from Maxma 60 rootstock translated into sweeter and flavorful cherries to the panelists. Other characteristics identified in routine analyses, such as color and firmness, went unnoticed by tasters (Figure 1).

### 3.2. Fruit Weight (FW) and Size (FS) 

**Figure 2:**
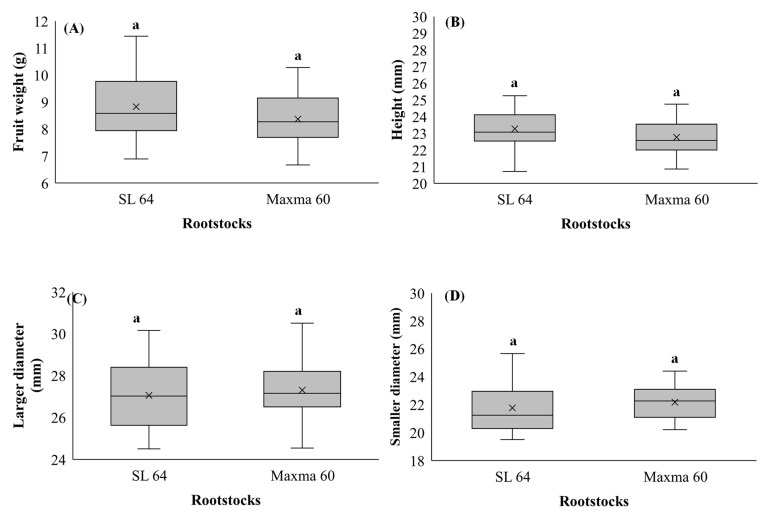
(**A**) Weight, (**B**) height, (**C**) larger diameter and (**D**) smaller diameter of sweet cherry cv. Early Bigi grafted on SL 64 and Maxma 60 rootstocks. Different letters indicate statistically significant differences (*p* < 0.05) according to Duncan’s test.

Fruit size is considered as the main benchmark used in commercial cherry grading, being a large factor in consumer preference, and is a huge determinant of both farm gate and market price. Fruit weight and size of sweet cherry cv. Early Bigi grafted on SL 64 and Maxma 60 rootstocks are presented in Figure 2. The average FW was 8.83 g for SL 64 and 8.37 g for Maxma 60. For the trees grafted on SL 64 rootstock, the average larger diameter was 27.04 mm, the average smaller diameter was 21.76 mm and the average height was 23.27 mm. For sweet cherries from trees grafted on Maxma 60 rootstock, the averages were 23.27 mm for height, 27.30 mm for larger diameter and 22.16 mm for the smaller diameter. No significant differences between rootstocks were observed in weight (*p* = 0.110), in larger diameter (*p* = 0.497), in smaller diameter (*p* = 0.282) or in height (*p* = 0.056). Positive correlations were observed between weight and all the other biometric parameters (R = 0.869, *p* < 0.001 for larger diameter; R = 0.689, *p* < 0.001 for smaller diameter; and R = 0.689, *p* < 0.001 for height).

According to Kappel et al. [69], the “ideal sweet cherry” should have an optimum fruit size, which is ≈ 12–15 g of weight and up to 34 mm of large diameter. In this work, no significant differences between rootstocks were observed in any biometric attributes. Additionally, the average weight and larger diameter were, in this study, lower than the values required for the “ideal sweet cherry”, for both rootstocks. This can be explained by the different environmental conditions and cultural practices. Indeed, Olmstead and Amy [70] reported that despite the mesocarp cell number being remarkably stable and virtually unaffected by the environment, i.e., growing location and physiological factors, the cell length was significantly affected by the environment, indicating that cultural practices that maximize mesocarp cell size should be used to achieve a cultivar’s fruit size potential.

In a study carried out by Szot and Meland [34], it was found that the type of rootstock significantly influences the external fruit properties, such as the size and percentage of stone in the total weight of the fruit. In another study, Forcada et al. [71] demonstrated that the scion–rootstock combination greatly influences some important attributes of the sweet cherry, such as vigor, yield and fruit size [43].

### 3.3. Fruit Color

Skin color is an important indicator of fruit maturity and quality, and consequently, it is a decisive consumer preference and acceptance parameter [72,73]. Chromatic parameters of sweet cherry cv. Early Bigi grafted on SL 64 and Maxma 60 rootstocks are presented in Table 1.

Lower C* values were obtained on Maxma 60 rootstock with a value of 36.47 which corresponded to a *hue***°** of 25.34, while for SL 64 a C* value of 41.34 was obtained, which corresponded to a *hue***°** of 34.26.

Significant differences between rootstocks were observed for all the chromatic parameters L*, a*, b*, C* and *hue***°** (*p* < 0.001, *p* < 0.05, *p* < 0.001, *p* < 0.001 and *p* < 0.001, respectively). All the chromatic parameters were positively correlated with each other (*p* < 0.001 for all the combinations, with exception of correlation between L* and a* that presented a *p* = 0.001).

The lowest chroma value obtained on cherries from trees grafted on Maxma 60 rootstock is indicative of an increase in the tonality of the fruit color, i.e., cherries with darker skin [63], with a significant decrease compared to the trees grafted on SL 64 rootstock. The fruits from these trees also presented the highest C* values, which is usually indicative of less mature cherries. Indeed, in general, the longer the fruit remains on the tree the redder it is [74], and the anticipation of the harvest of SL 64 rootstock may explain the higher chromatic parameters. Additionally, *hue*° and L* values were also lower in Maxma 60 rootstock, indicating redder and darker cherries. According to Gonçalves et al. [63], the reduction of L* indicates a loss of lightness, the photometric parameter proportional to the light reflected by the object. These findings are similar to those of Autio and Southwick [75] and Gonçalves et al. [43], who reported a significant effect of rootstock on the chromatic parameters of sweet cherry fruit. Milinović et al. [30] showed that rootstocks (‘Gisela 5’, ‘Gisela 6’, ‘PHL-C’ and ‘PiKU1’) had a strong influence on internal and external fruit quality parameters of cvs. ‘Kordia’ and ‘Regina’, finding correlations between some phenolics and color parameters. These authors also defend that the sweet cherry ripeness stage (harvest window) is also dependent on the rootstock.

### 3.4. Epidermis Rupture Force (ERF) and Flesh Firmness (FF)

According to Westwood et al. [76], the most common effects of rootstocks on fruit quality are differences in firmness and sugar content, which were also reported in the present work. However, for specific growing areas, studies are necessary to understand rootstock influence in each cultivar, to achieve favorable scion–rootstock combinations. In fact, fruit firmness is an important quality attribute in sweet cherries, which is associated with a greater resistance to decay and mechanical damage and, consequently, to the increase in storage life [77].

**Figure 3:**
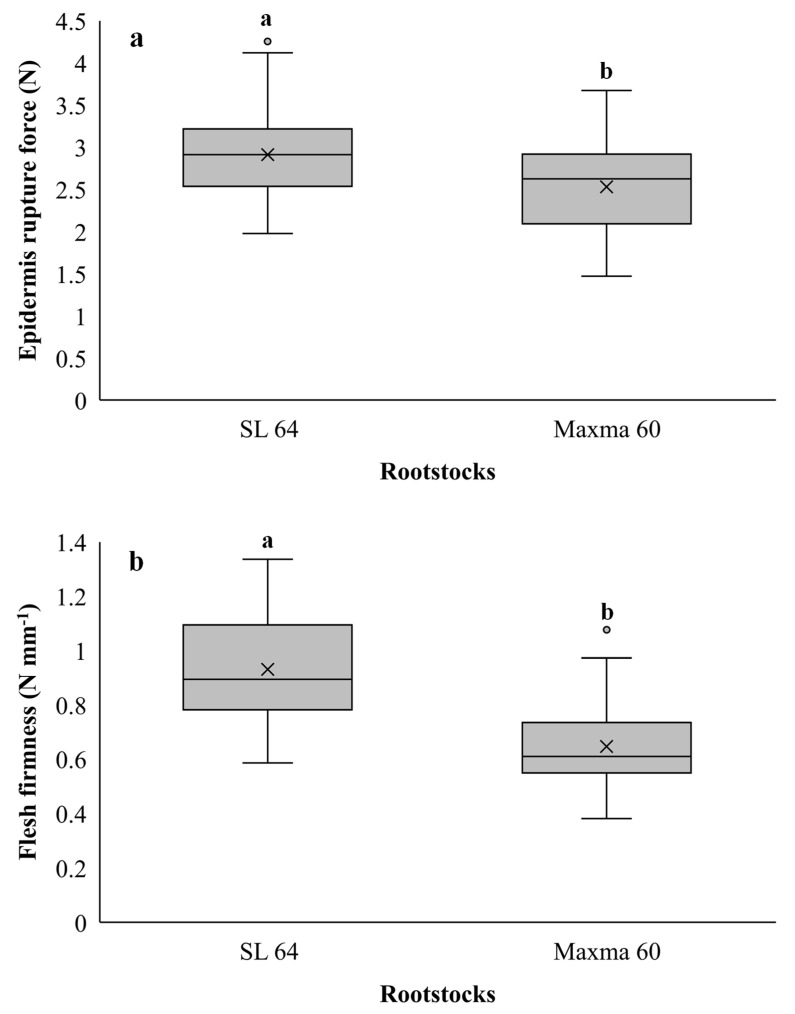
(**a**) Epidermis rupture force (ERF) and (**b**) flesh firmness (FF) of sweet cherry cv. Early Bigi grafted on SL 64 and Maxma 60 rootstocks. Different letters indicate statistically significant differences ( *p* < 0.05) according to Duncan’s test. The circles represent the outliers.

Regarding the texture parameters, the results of ERF and FF in sweet cherry cv. Early Bigi grafted on SL 64 and Maxma 60 rootstocks are presented in Figure 3.

Significant differences were observed in both parameters (*p* = 0.008 and *p* < 0.001 for ERF and FF, respectively). SL 64 rootstock presented fruits with the highest values for both parameters when compared to Maxma 60, consequently presenting fewer physiological disorders during handling, storage and shipping [78]. The two texture parameters presented the same profile and were positively correlated (R = 0.764, *p* < 0.001). Additionally, the lower texture parameters observed in fruits grafted on Maxma 60 were corroborated by the sensorial analysis, where the panelists considered that these fruits had a softer epidermis.

Several studies also demonstrated that the scion–rootstock combination influences the sweet cherry firmness [5,43,71].

### 3.5. Soluble Solids Content (SSC), Titratable Acidity (TA), Maturity Index (MI) and pH

The results of the maturity evaluation parameters of sweet cherry cv. Early Bigi grafted on SL 64 and Maxma 60 rootstocks are presented in Table 2. Regarding the SSC, cherries grafted on the rootstock Maxma 60 were sweeter (11.73 °Brix) and had lower titratable acidity (5.29% citric acid 100 g^−1^ of FW), consequently having a better maturation index (2.22) compared to SL 64 rootstock. According to López-Ortega et al. [79], the higher SSC is mainly due to a higher or more balanced fruit-to-leaf area ratio, usually characteristic of rootstocks with the lowest yields. Significant differences between rootstocks were observed for SSC, pH, TA and MI (*p* < 0.001 for each). Positive correlations were observed between SSC and pH (R = 0.309, *p* = 0.016), between SSC and TA (R = 0.665, *p* < 0.001) and between SSC and MI (R = 0.736, *p* < 0.001). On the other hand, TA was negatively correlated with pH (R = −0.430, *p* = 0.001) and with MI (R = −0.971, *p* < 0.001). The MI was simultaneously positively correlated with pH (R = 0.546, *p* < 0.001).

As the ratio between the SSC and the TA (sweetness/sourness) is considered an indicator of maturity [73,80], it is likely that fruits from trees grafted on SL 64 rootstock (MI = 1.91) were less mature and harvested a few days before full maturity than fruits from trees grafted on Maxma 60 rootstock (MI = 2.22). Indeed, as SL 64 rootstock allows anticipating the harvest concerning the Maxma 60 rootstock, sweet cherries from this scion–rootstock combination are some of the earliest collected in Resende region; they are sold very expensively, and sometimes producers tend to anticipate their harvest, which can lead to less ripe fruits.

The higher pH value observed in fruits from trees grafted on Maxma 60 can be related to the lower chromatic parameters observed in these fruits. This is in line with the findings of Gonçalves et al. [63], who related the decrease in color parameters of sweet cherry fruits with the increase in their pH.

In the study of Szot and Meland [34], it was shown that rootstock influences sweet cherry quality, namely in some internal characteristics such as quantity of soluble solids, titratable acidity and fruit juice pH. In another work developed by Forcada et al. [71], the scion–rootstock combination was also found to influence the SSC [43]. Furthermore, Dziedzic and Błaszczyk [5] also verified that the rootstock has an influence on the quality parameters of sweet cherry fruit after short-term storage, namely in SSC and TA.

### 3.6. Principal Component Analysis

**Figure 4:**
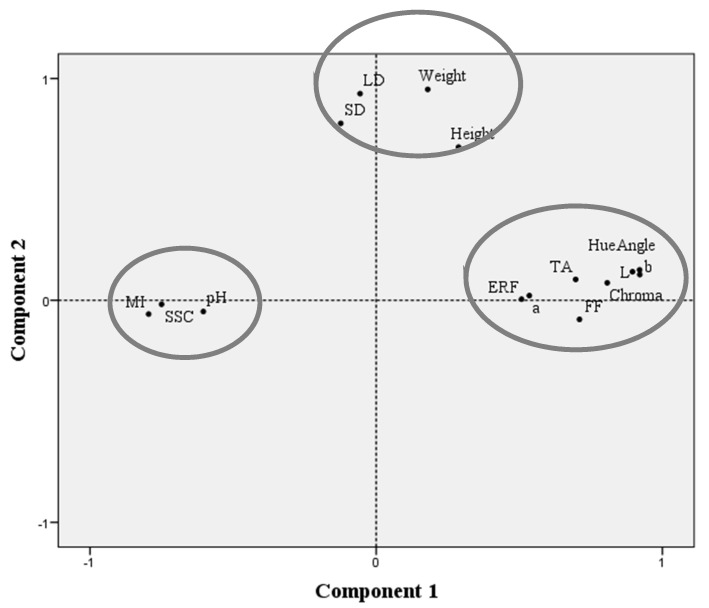
Principal component analysis using the whole dataset of sweet cherry Early Bigi grafted on SL 64 and Maxma 60 rootstocks. Analyzed parameters: biometric attributes (weight, height, larger diameter (LD) and smaller diameter (SD)); texture parameters (epidermis rupture force (ERF) and flesh firmness (FF)); maturity evaluation parameters (pH, titratable acidity (TA), soluble solids content (SSC) and maturity index (MI)); chromatic parameters (L*, a*, b*, chroma and *hue* angle).

To better understand the correlations between all the evaluated parameters, a chemometric analysis was performed integrating all the data (Figure 4). The PCA based on the correlation matrix standardizes the data, and this analysis was performed using a correlation matrix (Corr-PCA). In this analysis, the first two factorial axes (PC1 and PC2) represent 62.28% of the total variance, and factor 1 is the one that presents the higher weight (42.52%).

Taking into account the performed PCA, it was possible to observe three groups of variables perfectly separated in space: the first one included all the biometric parameters (weight, height, LD and SD were together between the left and the right PCA quadrants), the second one included all the chromatic and texture parameters (L*, a*, b*, Chroma, *hue*°, FF and ERF were together in the right quadrant) and the third one included the maturity parameters (SSC, MI and pH were spatially separated and placed in the left PCA quadrant). TA was an exception, which, due to its negative correlation with the remaining maturity parameters, was placed in the group of texture and color parameters, corroborating the negative correlations observed between pH and TA and between MI and TA.

### 3.7. Discriminant Analysis

**Figure 5:**
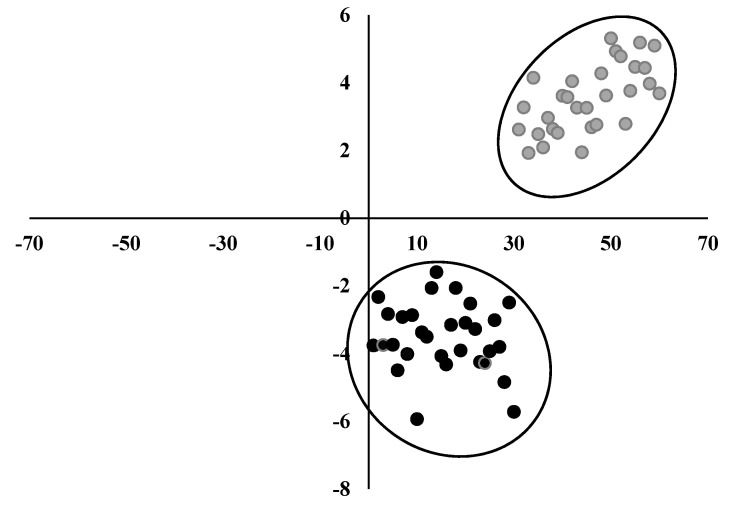
Discriminant canonical analysis of cultivars relative to quality parameters. Black dots represent fruits of cv. Early Bigi grafted on SL 64 rootstock, and gray dots represent fruits of cv. Early Bigi grafted on Maxma 60 rootstock.

To identify the quality parameters that better reflect the rootstock, a DCA was performed (Figure 5) globally showing that LD, SSC, pH, ERF, FF, a* and *hue*° mainly contribute to the distinction of the groups (Table 3). There was a separation of rootstocks along the second root according to the multivariate test statistics (Wilks’ lambda and corresponding F-value). In the present study, as shown in Table 3, seven variables constituted the best solution, and pH and FF were the variables that most contributed to the explained variation. These variables included in the function (Table 3) allowed explaining 100% of the data variability; i.e., by the function, all the analyzed fruits were placed in the right rootstock.

## 4. Conclusions

Studies involving the selection of the best scion–rootstock combination for each region are very important. In this work, the differences between trees grafted on SL 64 and Maxma 60 rootstocks were more evident in maturity, chromatic and texture parameters rather than in biometric attributes.

Indeed, trees grafted on Maxma 60 rootstock showed more mature fruits (higher SSC, MI and pH and lower TA). These results were also corroborated by the sensory profile analyzed by the panelists and by the lower chromatic parameters observed in this rootstock, which indicated darker and redder cherries. On the other hand, fruits from trees grafted on SL 64 rootstock presented better pulp firmness and, therefore, better texture, which is extremely useful for increasing the shelf life of this perishable fruit, although consumers prefer more mature fruits. However, as SL 64 rootstock allows anticipating the harvest in about one week compared with Maxma 60 rootstock, this scion–rootstock combination continues to be very profitable for the Resende region.

We conclude that the scion–rootstock combination is an important parameter to consider in orchard planting strategies since its influence in some attributes such as maturity, skin color and firmness of sweet cherries has been demonstrated in this study.

## Figures and Tables

**Table 1:** Chromatic parameters of sweet cherry cv. Early Bigi grafted on SL 64 and Maxma 60 rootstocks. Different letters indicate statistically significant differences (*p* < 0.05) according to Duncan’s test.

	L*	a*	b*	Chroma (C*)	*Hue* Angle (°)
**SL 64**	48.72 ± 4.96 a	34.13 ± 2.59 a	23.26 ± 2.30 a	41.34 ± 2.96 a	34.26 ± 2.61 a
**Maxma 60**	36.90 ± 3.20 b	32.82 ± 3.25 b	15.78 ± 3.58 b	36.47 ± 4.44 b	25.34 ± 3.01 b

**Table 2:** Soluble solids content (SSC) (°Brix), titratable acidity (TA) (g malicacid 100 g^−1^ of FW), maturity index (MI) (SSC/TA) and pH values of sweet cherry cv. Early Bigi grafted on SL 64 and Maxma 60 rootstocks. Different letters indicate statistically significant differences (*p* < 0.05) according to Duncan’s test.

	SSC	TA	MI	pH
**SL 64**	11.22 ± 0.42 b	6.03 ± 1.00 a	1.91 ± 0.27 b	3.79 ± 0.04 b
**Maxma 60**	11.73 ± 0.15 a	5.29 ± 0.26 b	2.22 ± 0.12 a	3.85 ± 0.04 a

**Table 3:** Discriminant analysis using the whole dataset of sweet cherry Early Bigi grafted on SL 64 and Maxma 60 rootstocks. Parameters included in the function: larger diameter (LD), soluble solids content (SSC), pH, epidermis rupture force (ERF), flesh firmness (FF), a* and *hue* angle.

Wilks’ Lambda				
Function test	Wilks’ lambda	Chi-square	Gl	Sig.
1	0.094	128.968	7	0.000
**Eigenvalues**				
Function	Eigenvalue	% of variance	Cumulative %	Canonical correlation
1	9.659 ^a^	100.0	100.0	0.952
a. The first canonical discriminating function was used in the analysis.				
**Canonical discriminant function coefficients**				
		Function		
		1		
LD		0.217		
SSC		1.252		
pH		13.676		
ERF		1.079		
FF		−4.830		
a*		0.221		
*Hue* angle		−0.442		
(Constant)		−65.835		

## Data Availability

Not applicable.
